# Rapamycin improves healthspan but not inflammaging in *nfκb1* ^−/−^ mice

**DOI:** 10.1111/acel.12882

**Published:** 2018-11-23

**Authors:** Clara Correia‐Melo, Jodie Birch, Edward Fielder, Dina Rahmatika, Jennifer Taylor, James Chapman, Anthony Lagnado, Bernadette M. Carroll, Satomi Miwa, Gavin Richardson, Diana Jurk, Fiona Oakley, Jelena Mann, Derek A. Mann, Viktor I. Korolchuk, João F. Passos

**Affiliations:** ^1^ Newcastle University Institute for Ageing, Institute for Cell and Molecular Biosciences Newcastle University Newcastle upon Tyne UK; ^2^ Department of Physiology and Biomedical Engineering Mayo Clinic Rochester Minnesota; ^3^ Cardiovascular Research Centre, Institute of Genetic Medicine, International Centre for Life Newcastle University Newcastle upon Tyne UK; ^4^ Faculty of Medical Sciences, Institute of Cellular Medicine Newcastle University Newcastle upon Tyne UK; ^5^ Present address: The Molecular Biology of Metabolism Laboratory The Francis Crick Institute London UK; ^6^ Present address: Department of Biochemistry University of Cambridge Cambridge UK; ^7^ Present address: London Institute of Medical Sciences Imperial College London London UK

**Keywords:** aging, inflammaging, mTOR, rapamycin, SASP, senescence

## Abstract

Increased activation of the major pro‐inflammatory NF‐κB pathway leads to numerous age‐related diseases, including chronic liver disease (CLD). Rapamycin, an inhibitor of mTOR, extends lifespan and healthspan, potentially via suppression of inflammaging, a process which is partially dependent on NF‐κB signalling. However, it is unknown if rapamycin has beneficial effects in the context of compromised NF‐κB signalling, such as that which occurs in several age‐related chronic diseases. In this study, we investigated whether rapamycin could ameliorate age‐associated phenotypes in a mouse model of genetically enhanced NF‐κB activity (*nfκb1* ^−/−^) characterized by low‐grade chronic inflammation, accelerated aging and CLD. We found that, despite showing no beneficial effects in lifespan and inflammaging, rapamycin reduced frailty and improved long‐term memory, neuromuscular coordination and tissue architecture. Importantly, markers of cellular senescence, a known driver of age‐related pathology, were alleviated in rapamycin‐fed animals. Our results indicate that, in conditions of genetically enhanced NF‐κB, rapamycin delays aging phenotypes and improves healthspan uncoupled from its role as a suppressor of inflammation.

## 1 INTRODUCTION

Chronic inflammation is a major factor underpinning aging and major age‐related diseases, a process known as inflammaging (Franceschi et al., 2007). Misregulation of the NF‐κB pathway, a major regulator of inflammaging, occurs in numerous age‐associated diseases, including cancer (Courtois & Gilmore, 2006).

NF‐κB also plays a key role in cellular senescence, a state of irreversible cell‐cycle arrest induced by various forms of cellular stress, acting as a master regulator of the senescence‐associated secretory phenotype (SASP) (Chien et al., 2011). The SASP can have beneficial effects such as the facilitation of immune surveillance and the clearance of senescent cells (Kang et al., 2011). However, an age‐dependent accumulation of senescent cells resulting in a chronic SASP is thought to play a causal role in age‐related pathology and cancer induction (Birch & Passos, 2017).

The NF‐κB signalling is tightly associated with the mechanistic target of rapamycin (mTOR) pathway to modulate age‐ and senescence‐associated phenotypes. mTOR is a key modulator of lifespan, as evidenced by increased longevity in a range of model organisms, from yeast to mice, upon its inhibition (Johnson, Rabinovitch, & Kaeberlein, 2013a). The drug rapamycin is an inhibitor of mTOR complex 1 (mTORC1) and increases lifespan and delays the onset of age‐related pathologies in mice (Harrison et al., 2009). Rapamycin is also a key inhibitor of senescence and the SASP at multiple levels: Rapamycin can inhibit the translation of IL‐1α, a key regulator of the SASP (Laberge et al., 2015) and of MK2 kinase which controls the stability of mRNA encoding for several SASP components (Herranz et al., 2015). Moreover, rapamycin can inhibit mitochondrial ROS‐induced NF‐κB activation during senescence (Correia‐Melo et al., 2016). Importantly, rapamycin has been shown to suppress the tumour‐promoting abilities of senescent cells (Herranz et al., 2015; Laberge et al., 2015), suggesting that some of the beneficial effects of rapamycin in vivo are partly mediated by its ability to act as a SASP suppressor, limiting inflammaging and cancer development.

While evidence indicates that rapamycin ameliorates inflammatory phenotypes and improves a wide range of healthspan traits during aging in the absence of enforced inflammation, the effectiveness of rapamycin in conditions where NF‐κB signalling is compromised such as during several age‐related diseases is still unknown.

In this study, we investigated the impact of rapamycin treatment in a mouse model showing premature aging phenotypes driven by genetically enhanced NF‐κB activation, the *nfκb1* knockout (*nfκb1* ^−/−^) mouse (Jurk et al., 2014; Wilson et al., 2015). *nfκb1* ^−/−^ mice have increased inflammation due to the absence of the NF‐κB1 (p50) subunit, which under normal conditions acts as a suppressor of NF‐κB activity by competing with RelA (the classic pro‐inflammatory NF‐κB subunit)‐containing dimers and by recruiting histone deacetylase 1 (HDAC1) which tightly packs DNA causing the active repression of transcription (Cartwright, Perkins, & C LW, 2016).

Here, we found that lifelong treatment with rapamycin does not impact on the lifespan of male *nfκb1* ^−/−^ mice, but improves healthspan, including long‐term memory, neuromuscular coordination and forelimb grip strength, and reduces overall frailty. Moreover, age‐associated phenotypes such as increased cardiac hypertrophy, lung emphysema and skin epidermal thinning observed in *nfκb1* ^−/−^ mice were ameliorated following rapamycin supplementation. While rapamycin decreased senescent markers in *nfκb1* ^−/−^ mice, no significant reduction in the pro‐inflammatory phenotype was observed. Our findings suggest that under conditions of genetically enhanced NF‐κB activation, rapamycin results in beneficial health effects uncoupled from its role as a suppressor of inflammation.

## 2 RESULTS

### 2.1 Rapamycin improves overall healthspan in *nfκb1* ^−/−^ mice

**Figure 1:**
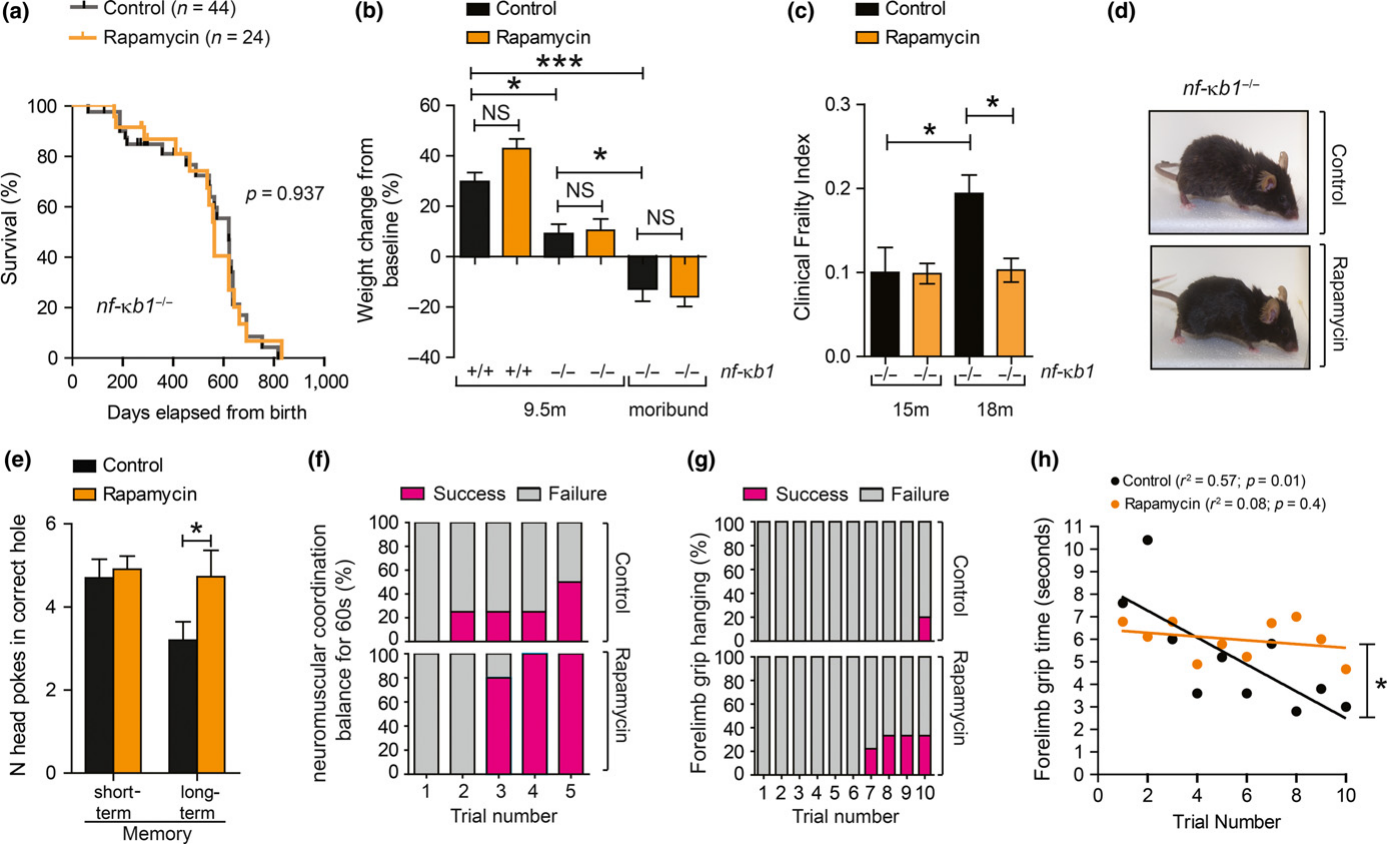
Rapamycin prevents age‐related frailty in *nf‐κb1* ^−/−^ mice without impacting on lifespan. (a) Kaplan–Meier survival curves of *nf‐κb1* ^−/−^ mice fed with a control (*n* = 44) or rapamycin‐supplemented diet (*n* = 24), from 4 to 5 months old until death; (b) percentage of body weight change from baseline (start of diet) in control or rapamycin‐fed *nf‐κb1* ^−/−^ mice at 9.5 months and in moribund animals (*n* = 7–8 per group); (c) Clinical Frailty Index at 15 and 18 months of age in *nf‐κb1* ^−/−^ with or without rapamycin diet (*n* = 8–11 per group); (d) representative images of *nf‐κb1* ^−/−^ mice with or without rapamycin feeding at 18 months of age; (e) Barnes maze test in *nf‐κb1* ^−/−^ mice with or without rapamycin diet (*n* = 9 per group); (f) neuromuscular coordination measured as % number of successful attempts (in purple) to remain on a straight rod for 60 s (*n* = 4–5 per group; 9.5 months old); (g) forelimb grip strength measured as number of trials required to remain hanging for total of 90 s (% success in purple (*n* = 5–9 per group; 18 months old); (h) linear regression of mean forelimb grip time (seconds). All data are mean ± *SEM*. *p < 005, **p < 0.01, ***p < 0.001 (One‐way ANOVA)

To determine whether mTOR inhibition improves lifespan or healthspan in a mouse model of genetically enhanced NF‐κB activity, we fed *nfκb1* ^−/−^ mice a control or rapamycin‐supplemented diet, as previously described (Harrison et al., 2009). *nfκb1* ^−/−^ mice fed rapamycin showed no significant differences in mean and maximum lifespan (Figure 1a) or in body weight throughout their life (Figure 1b) when compared to mice fed a control diet. However, rapamycin resulted in significant improvements in multiple parameters of clinical frailty (Whitehead et al., 2014) with age (Figure 1c,d and Supporting Information Table S1). Other health parameters known to decline with age in both wild‐type (wt) and *nfκb1* ^−/−^ mice were improved upon rapamycin feeding, such as long‐term memory (Figure 1e), neuromuscular coordination (Figure 1f) and forelimb grip strength (Figure 1g,h).

**Figure 2:**
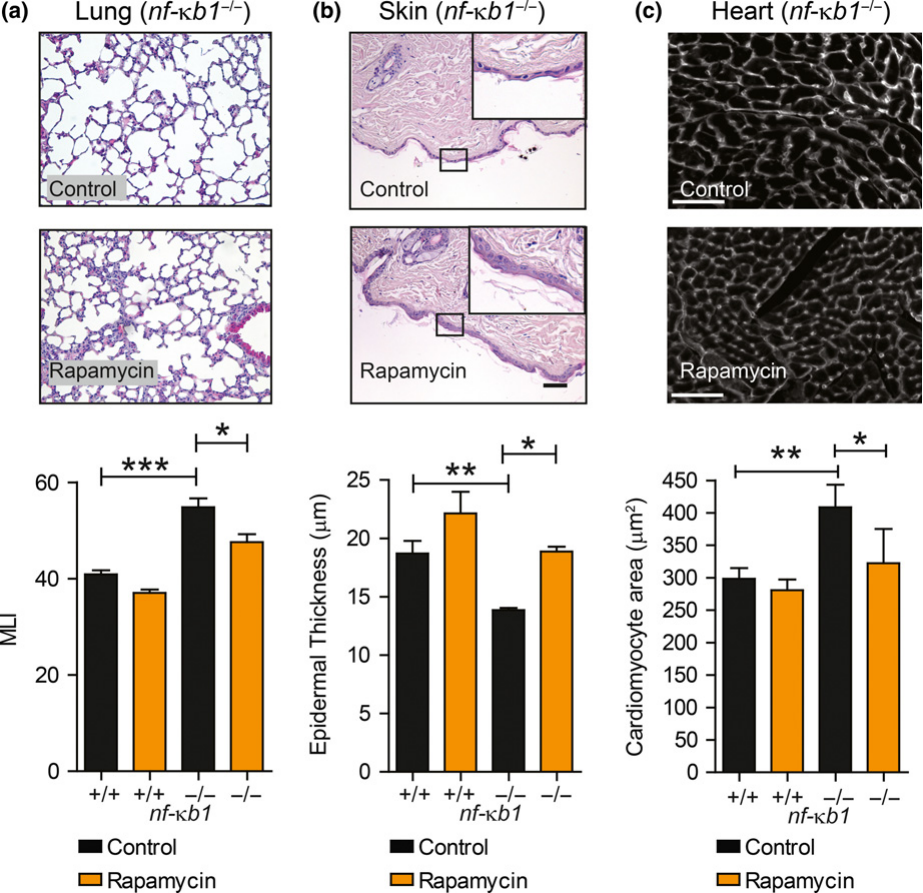
Rapamycin ameliorates several aging‐associated histological parameters in *nf‐κb1* ^−/−^ mice. (a) Representative images of H&E staining of lung tissue sections from *nf‐κb1* ^−/−^ mice fed a control or rapamycin‐supplemented diet with quantifications of mean linear intercept (MLI) at 9.5 months of age; (b) representative images of H&E staining of skin sections from 9.5‐month‐old *nf‐κb1* ^−/−^ mice fed with control or rapamycin‐supplemented diet plus quantifications of mean epidermal thickness; (c) representative images of heart sections labelled with wheat germ agglutinin WGA from 9.5‐month‐old *nf‐κb1* ^−/−^ mice fed with control or rapamycin‐supplemented diet plus quantifications of cross‐sectional cardiomyocyte area. All data are mean ± *SEM* (*n* = 4–8 mice per group). *p < 005, **p < 0.01, ***p < 0.001 (One‐way ANOVA)

Lung airspace enlargement, indicative of emphysematous‐like changes, epidermal thinning of the skin and increased cardiac hypertrophy which occur during normal aging and prematurely in *nfκb1* ^−/−^ mice, was significantly reduced following a rapamycin treatment (Figure 2a–c). *nfκb1* ^−/−^ mice develop chronic liver disease (CLD), characterized by the emergence of dysplastic nodules, fibrosis and increased tumour frequency (Wilson et al., 2015). Histopathological analysis of livers from *nfκb1* ^−/−^ mice at 9.5 months of age did not show fibrosis (Supporting Information Table S2), as confirmed by α‐SMA and Sirius red analysis (Supporting Information Figure S1). However, at later stages, moribund (debilitating state immediately prior to death) *nfκb1* ^−/−^ mice showed evidence of CLD, characterized by enhanced fibrosis and dysplasia, which was not reduced by rapamycin (Supporting Information Table S2).

### 2.2 Rapamycin does not alleviate inflammation in *nfκb1* ^−/−^ mice

**Figure 3:**
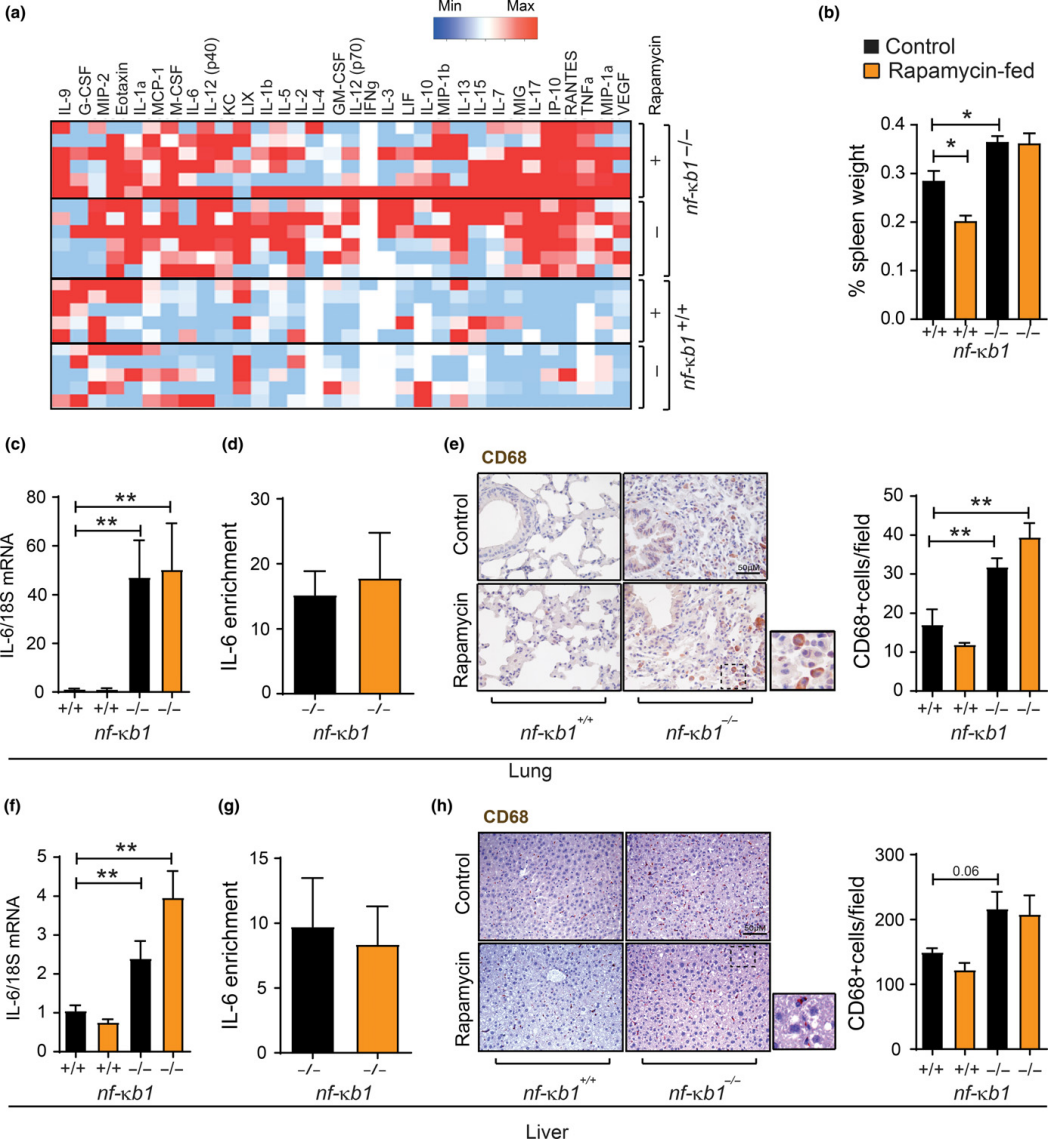
Rapamycin does not reduce inflammation in *nf‐κb1* ^−/−^ mice. (a) Serum cytokine array from wild‐type (*n* = 5) or *nfκb1* ^−/−^ mice (*n* = 6) fed a control (−) or rapamycin‐supplemented (+) diet; (b) spleen weight, represented as percentage of total body weight in wild‐type or *nfκb1* ^−/−^ mice fed control or rapamycin‐supplemented diet at 9.5 months of age; (c) IL‐6 mRNA levels in whole lung, or liver (f), tissue from wild‐type or *nfκb1* ^−/−^ mice fed control or rapamycin‐supplemented diet, normalized to 18S; (d) ChIP analysis of RELA enrichment at the IL‐6 promoter in whole lung, or liver (g), from *nfκb1* ^−/−^ mice fed a control or rapamycin‐supplemented diet; (e) and (h) representative images of CD68 immunohistochemical staining in lung or liver tissue sections, respectively, from wild‐type or *nf‐κb1* ^−/−^ mice fed with control or rapamycin‐supplemented diet plus quantifications of CD68+ cells/field. Data represent group mean ± *SEM* (*n* = 3–7 mice per group). *p < 005, **p < 0.01, ***p < 0.001 (One‐way ANOVA)

To determine whether mTOR inhibition by rapamycin reduces chronic inflammation in *nfκb1* ^−/−^ mice, we analysed serum cytokine levels from wt and *nfκb1* ^−/−^ mice fed a control or rapamycin‐supplemented diet. While a vast array of pro‐inflammatory mediators, including Interleukin‐6 (IL‐6), tumour necrosis factor‐alpha and vascular endothelial growth factor, was increased in the serum from *nfκb1* ^−/−^ mice, rapamycin had no significant effect on these factors (Figure 3a). Spleen enlargement, another indicator of inflammation (Jurk et al., 2014), was also unchanged upon rapamycin treatment in* nfκb1* ^−/−^ mice (Figure 3b). In addition, rapamycin did not alter the expression of IL‐6 at the mRNA level as well as binding of the NF‐κB subunit RelA (p65) to the IL‐6 promoter in the lungs and livers of *nfκb1* ^−/−^ mice (Figure 3c,d,f,g). Furthermore, *nfκb1* ^−/−^ mice showed an increased number of CD68‐positive cells, a macrophage marker, in the liver and lungs; however, there was no significant effect of a rapamycin‐supplemented diet (Figure 3e,h).

These results indicate that mTOR inhibition with rapamycin does not alleviate inflammation in a mouse model of genetically enhanced NF‐κB activity.

### 2.3 Rapamycin alleviates markers of senescence and mitochondrial dysfunction in *nfκb1* ^−/−^ mice

**Figure 4:**
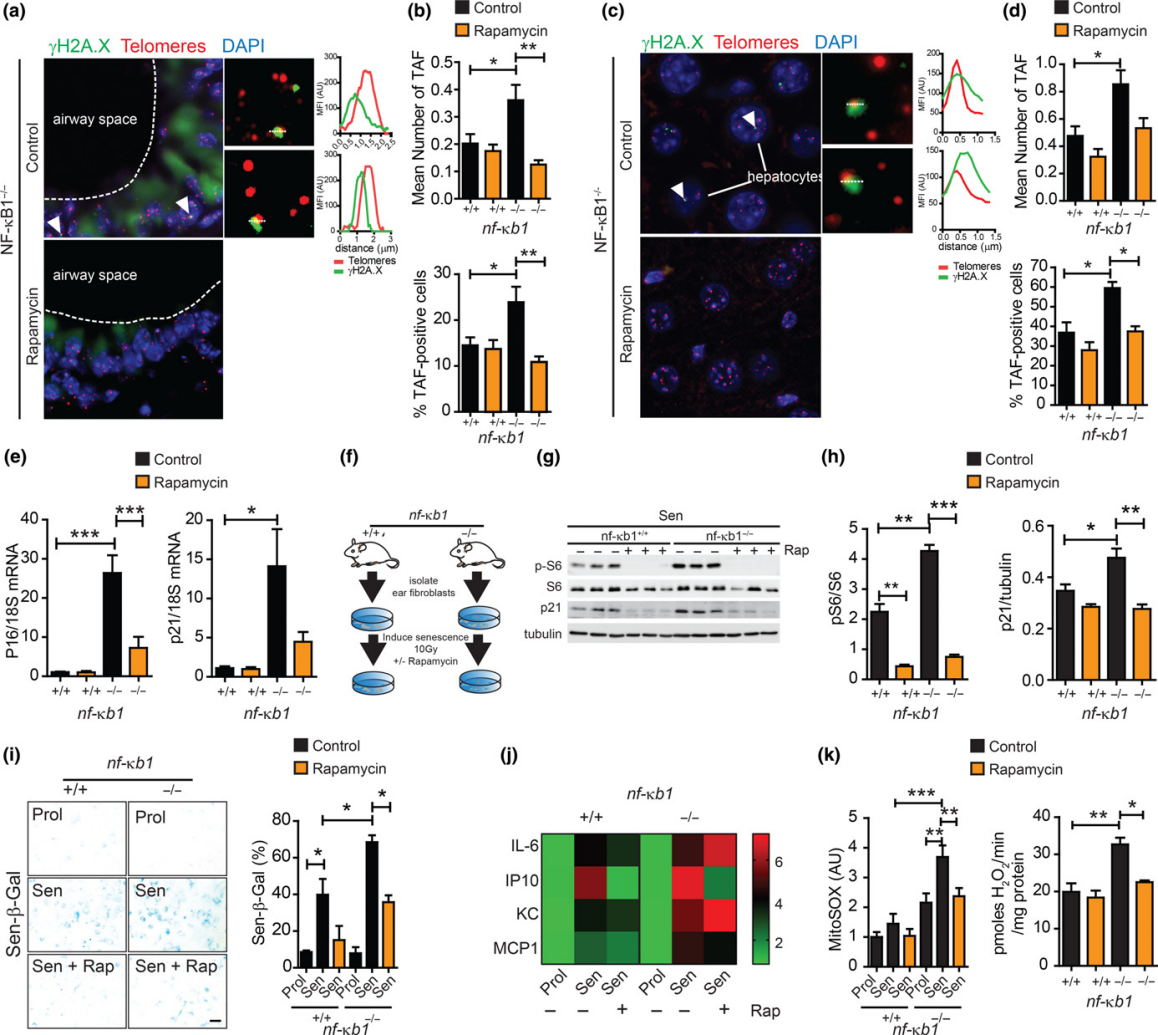
Rapamycin reduces markers associated with cellular senescence and mitochondrial dysfunction in vivo and in vitro. (a) Representative images of immunofluorescence in situ hybridization (immuno‐FISH) staining in lung airway epithelium, or hepatocytes in liver sections (c) of *nfκb1* ^−/−^ mice fed control or rapamycin‐supplemented diet at 9.5 months of age. Images are maximum intensity projections of at least 50 planes with areas of colocalization in single Z planes shown on the right. Graphs represent quantification of γH2A.X and telomere signals in selected regions of interest (dotted lines); (b) frequencies of telomere‐associated foci (TAF) in small airway epithelial cells, or hepatocytes (d), from 9.5‐month‐old wild‐type and *nfκb1* ^−/−^ mice fed a control or rapamycin‐supplemented diet; (e) p16 and p21 mRNA levels in whole lung tissue from wild‐type or *nfκb1* ^−/−^ mice (9.5 months old) fed control or rapamycin‐supplemented diet, normalized to 18S; (f) mouse adult fibroblasts (MAFs) isolated from the ears of wt or *nfκb1* ^−/−^ mice were induced to senescence by 10 Gy irradiation and cultured in the presence or absence of rapamycin at 3% O_2_; (g) representative western blot showing phospho‐S6 (pS6), S6 and p21 expression in senescent MAFs from wild‐type and *nfκb1* ^−/−^ mice treated with rapamycin (+) or DMSO (−) with quantifications on the right (h); (i) representative images of senescence‐associated β‐galactosidase staining in MAFs from wild‐type or *nfκb1* ^−/−^ mice treated with rapamycin (+) or DMSO (−) with quantifications of percentage positive cells shown on the right; (j) heatmap depicting fold change in indicated cytokines in medium of proliferating and senescent MAFs from wild‐type and *nfκb1* ^−/−^ mice treated with or without rapamycin measured using a Multiplexing LASER Bead Assay; (k) mitochondrial ROS (MitoSOX) levels in proliferating and senescent MAFs from wild‐type and *nfκb1* ^−/−^ mice treated with or without rapamycin and hydrogen peroxide generation in isolated mitochondria from the livers of wild‐type and *nfκb1* ^−/−^ mice (9.5 months old) fed a control or rapamycin‐supplemented diet. Data are mean ± *SEM* (*n* = 3–7 mice per group for all analyses). *p < 005, **p < 0.01, ***p < 0.001 (One‐way ANOVA)

Telomere‐associated DNA damage foci (TAF) are established markers of cellular senescence (Hewitt et al., 2012). Furthermore, increased frequency of TAF occurs in tissues from *nfκb1* ^−/−^ mice when compared to wild‐type (Jurk et al., 2014). To determine whether mTOR inhibition with rapamycin affects telomere dysfunction in *nfκb1* ^−/−^ mice, we analysed TAF in lung and liver tissues from *nfκb1* ^−/−^ mice fed a control or rapamycin‐supplemented diet. We report a significant increase in the mean number of TAF and percentage of TAF‐positive lung airway epithelial cells in *nfκb1* ^−/−^ mice, which was reduced by rapamycin (Figure 4a,b). We have previously reported that TAF increase in hepatocytes with age in wt (Hewitt et al., 2012) and *nfκb1* ^−/−^ mice (Jurk et al., 2014) and can be reduced by pharmacogenetic elimination of p16^Ink4a^‐positive senescent cells (Ogrodnik et al., 2017). We observed that the increase in mean number of TAF and percentage of TAF‐positive hepatocytes in *nfκb1* ^−/−^ mice compared to controls was suppressed by rapamycin (Figure 4c,d).

Telomere dysfunction is an important driver of cellular senescence in vivo and in vitro (Hewitt et al., 2012). Accordingly, we analysed markers of senescence, downstream of telomere dysfunction in lung tissue, which shows an age‐dependent accumulation of senescent cells and has increased frequency of senescent cells under conditions of chronic inflammation (Birch et al., 2015; Schafer et al., 2017). mRNA expression of the cyclin‐dependent kinase inhibitors p16 and p21 was significantly increased in whole lungs of *nfκb1* ^−/−^ compared to wild‐type mice and was reduced by rapamycin (Figure 4e).

To further determine whether rapamycin impacts on the development of senescence in this model of chronic inflammation, we induced senescence in mouse adult fibroblasts (MAFs), isolated from wt or *nfκb1* ^−/−^ mice, by ionizing radiation (IR) and treated them with rapamycin (Figure 4f). MAFs isolated from *nfκb1* ^−/−^ mice showed increased mTOR activity as compared to wt (determined by phosphorylated S6 to total S6 ratio), which was reduced following rapamycin treatment (Figure 4g). In agreement with previous observations (Jurk et al., 2014), MAFs from *nfκb1* ^−/−^ mice showed an enhanced senescence response to IR as compared to wt, evidenced by increased senescence‐associated β‐galactosidase (Sen‐β‐Gal) activity and expression of p21 and p16. These markers were significantly reduced upon rapamycin treatment in MAFs derived from both wt and* nfκb1* ^−/−^ mice (Figure 4g–i and Supporting Information Figure S2). Despite reduced expression of the cyclin‐dependent kinase inhibitors p21 and p16, there was no rescue of proliferation (determined by Ki67 positivity) by rapamycin following senescence induction in wt and *nfκb1* ^−/−^ MAFs, consistent with previous reports (Correia‐Melo et al., 2016) (Supporting Information Figure S2). Senescent *nfκb1* ^−/−^ MAFs showed a significant increase in the secretion of SASP‐associated components IL‐6, IP‐10, KC and MCP1, when compared to senescent wt MAFs and remained unchanged in *nfκb1* ^−/−^ MAFs upon rapamycin treatment (Figure 4j).

It has been proposed that *nfκb1* ^−/−^ mice have accelerated aging and senescence phenotypes, likely due to increased mitochondrial ROS production leading to telomere dysfunction (Jurk et al., 2014). Furthermore, rapamycin can reduce mitochondrial ROS generation and TAF in livers of aged wt mice (Correia‐Melo et al., 2016) and improves the survival and healthspan in a mouse model of Leigh syndrome, where mitochondria complex I activity is impaired (Johnson, Yanos, et al., 2013b). Therefore, we sought to determine whether mTOR inhibition with rapamycin impacts on mitochondrial ROS generation in the context of enforced NF‐κB activity. We found that senescent MAFs from *nfκb1* ^−/−^ mice showed enhanced production of mitochondrial‐derived ROS, as compared to MAFs from wt mice, which was significantly reduced with rapamycin. Furthermore, isolated mitochondria from livers of *nfκb1* ^−/−^ mice produced increased ROS when compared to age‐matched wt mice, which could be alleviated by rapamycin treatment (Figure 4k).

## 3 DISCUSSION

Previous studies have demonstrated that rapamycin treatment extends lifespan and delays the onset of a number of age‐related pathologies in wt mice (Harrison et al., 2009; Houssaini et al., 2018; Lesniewski et al., 2017; Reifsnyder, Flurkey, Te, & Harrison, 2016), possibly by reducing the tumour‐promoting abilities of senescent cells, particularly the SASP (Herranz et al., 2015; Laberge et al., 2015). Here, we aimed to understand the impact of rapamycin in the context of enforced deregulation of the NF‐κB pathway, a major regulator of aging and cancer. We report that rapamycin does not rescue the shortened lifespan and CLD in *nfκb1* ^−/−^ mice, which we speculate may be due to its inefficiency in counteracting inflammaging.

Nevertheless, rapamycin reduced a range of shared aging and senescence‐associated markers. These observations are supported by the fact that the link between chronic inflammation and aging is, at least in part, driven by the accumulation of senescent cells, as evidenced by the delayed onset of age‐related inflammatory pathologies and improved healthspan upon elimination of senescent cells in wild‐type mice and models of age‐related disease (Baker et al., 2016; Ogrodnik et al., 2017; Schafer et al., 2017; Xu et al., 2018) and the increased frequencies of senescent cells in the *nfκb1* ^−/−^ mouse (Jurk et al., 2014). Indeed, there is increasing interest in finding therapies to eliminate senescent cells or target specifically the deleterious aspects of the senescent phenotype, including its pro‐inflammatory and pro‐oxidant components (Birch & Passos, 2017).

We also report a reduction in mitochondrial ROS by rapamycin treatment both in vitro and in mitochondria isolated from mouse livers, coupled to a reduction in TAF in *nfκb1* ^−/−^ mice. This together with the fact that: (a) rapamycin can reduce mitochondrial ROS (Miwa et al., 2014; Correia‐Melo et al., 2016); (b) mitochondrial ROS can induce telomere dysfunction and accelerate senescence (Passos et al., 2007); and (c) in addition to the paracrine effects of the SASP, ROS leaked by senescent cells can also induce senescence in neighbouring cells (Nelson et al., 2012), leading to the hypothesis that improvements in mitochondrial function may contribute to reduced activation of cellular senescence regulating pathways, which could account for the improved overall healthspan in the *nfκb1* ^−/−^ mice.

Another potential mechanism by which rapamycin improves healthspan is by preventing stem cell dysfunction. NF‐κb1^−/−^ mice have been shown to exhibit stem cell dysfunction and impaired regeneration (Jurk et al., 2014). Interestingly, stem cell depletion in mice has recently been shown to causally contribute to senescence induction and premature aging phenotypes (Vilas et al., 2018). Furthermore, mTOR has been shown to negatively impact on stem cell function potentially via mitochondrial dysfunction and ROS generation (Chen et al., 2008; Sousa‐Victor et al., 2014). Thus, it is possible that rapamycin prevents stem cell decline in NF‐kB1^−/−^ despite chronic inflammation.

It should be noted that our study was limited to male mice, and with the same doses of rapamycin, effects on survival were stronger in females than males (Harrison et al., 2009); thus, future investigations should account for gender effects.

In summary, our results show that rapamycin can ameliorate certain age‐related phenotypes irrespectively of the inflammatory burden. Given the known tight relationship between inflammaging, dysfunctional NF‐κB signalling and several age‐related diseases, our results may have important clinical and conceptual implications.

## 4 EXPERIMENTAL PROCEDURES

### 4.1 Mice groups, treatments and housing

Experiments were performed on male *nf‐κb1* ^−/−^ mice on a pure C57Bl/6 background and C57Bl/6 wild‐type controls. Mice were split into two groups according to age and diet: (a) 9.5‐month‐old mice fed a control or rapamycin‐supplemented diet (as described in Harrison et al., 2009) for 6 months prior to humane end point and (b) lifespan mice (*nf‐κb1* ^−/−^ mice only) fed with a control or rapamycin‐supplemented diet from 4 to 5 months old until moribund state followed by humane end points. The different mice groups were matched for age and randomly assigned for the treatments. Control and rapamycin diets were purchase from TestDiet—control diet: 5LG6/122 PPM EUDRAGIT 3/8 #1814831 (5AS0) and encapsulated rapamycin diet: 5LG6/122 PPM ENCAP RAP 3/8 #1814830 (5ARZ). Mice were housed in cages (56 cm_38 cm_18 cm, North Kent Plastics, Kent, UK) of groups of 4–6 that did not change from weaning. Mice had ad libitum (AL) access to food and water and were monitored weekly. Mice were housed at 20 ± 2°C under a 12 hr light/12 hr dark photoperiod with lights on at 07:00 hr.

Maximum lifespan was estimated as the lifespan of the longest living 1%–3% of the cohort. Medians were calculated from right‐censored Kaplan–Meier curves. Ethical approval was granted by the LERC Newcastle University, UK. All experiments were undertaken in compliance with UK Home Office legislation under the Animals (Scientific Procedures) Act 1986. No statistical method was used to predetermine sample size. No animals or samples were excluded from the analysis.

Organs and tissues were collected during necropsy and fixed with either 10% formalin (VWR; 9713.9010) and paraffin embedding for histochemical analysis or snap‐frozen in liquid nitrogen and stored at −80°C for biochemical analysis.

### 4.2 Frailty measurements

Frailty was assessed in *nfκb1* ^−/−^ mice using the Clinical Frailty Index described by Whitehead et al. (2014). Body weight and temperature are given as mean values and are not included in the sum frailty index score. Mice were scored 0 for no frailty phenotype, 0.5 for mild phenotype and 1 for severe phenotype. This was performed simultaneously by two trained assessors, including an experienced animal technician. Clinical examination was performed at the same time of the day each time. Mice were observed in their home cage, before being moved to a sterile hood. Body weight was recorded, and surface temperature was measured using an infrared temperature probe (Raynger MX, Raytek).

### 4.3 Short‐ and long‐term memory tests

Short‐ and long‐term memory was assessed in *nfκb1* ^−/−^ mice using a Barnes maze test. The maze consists of an open circular surface (diameter = 92 cm), with a series of 20 holes (diameter = 5 cm) along the border and four visual markers at 0°, 90°, 180° and 270° positions (square, cross, triangle or circle). These visual markers are placed on the walls around the maze, and the maze is elevated so that mice cannot exit via the sides. Under one of these holes is attached the “target box,” which the mouse can enter and provide refuge from the bright and exposed open surface top. The experiment requires the subject to learn the spatial position of this escape box relative to a number of visual cues positioned on the surrounding walls. The mouse is placed in an opaque pipe (diameter = 11 cm, height = 15 cm), in the centre of the maze and allowed 10 s to calm. The pipe is removed, and the timer started. An overhead camera, linked to a DVD recorder and monitor, allows the performance of the mouse to be observed and recorded. Each mouse is assigned one to one the four visual markers, under which the escape box will be placed during training. In initial run, naïve mice are guided to the escape hole and allowed to rest inside for 2 min to acclimatize. The “Acquisition phase” follows this, with mice running four trials per day, for 4 days. In each trial, the mouse is given 3 min to find and enter the escape hole. If the mouse does not find the hole in the allotted time then it is again guided to the escape hole. The mouse is allowed to rest in the target box for 1 min. Between trials, the board and escape box are wiped with 70% EtOH to remove odour trails. Short‐term memory was tested 24 hr after training on day 5 in a 90 s trial, without the target box present. Long‐term retention is tested in the same fashion 12 days after training, with no training between these trials. The number of times the mouse moves its head over, or into, the target hole was recorded during these trials was recorded.

### 4.4 Forelimb grip strength analysis

Forelimb grip strength was assessed by allowing animals to grip a suspended wire coat hanger with a diameter of 2 mm and a length of 30 cm using its forelimbs. Time spent hanging from the wire following grip was recorded across 10 attempts up to a total of 90 s. Completion was defined as having succeeded in hanging for a total sum of 90 s. The test was terminated after 90 s or once 10 trials were performed. Trials were defined as failures if the animal fell from the wire, after which the timer was paused and the replaced after a 20‐s resting period.

### 4.5 Neuromuscular coordination analysis

Neuromuscular coordination was assessed using the tightrope test (Jurk et al., 2014). Briefly, animals were placed on a horizontal bar, with a diameter of 1.5 cm and a length of 60 cm, and time spent on top of the bar was recorded. A trial was considered successful if the animal could remain on the bar for 60 s without falling. Each mouse was given five consecutive trials.

### 4.6 Mouse adult fibroblast (MAFs) culture

MAFs were isolated from ear clippings from wild‐type and *nfκb1* ^−/−^ mice and cultured as previously described (Ogrodnik et al., 2017). Each primary cell line was derived from a separate donor. MAFs were seeded and allowed to grow for 24 hr, and then, X‐ray irradiated with 10 Gy using a PXI X‐Rad 225 (RPS Services Ltd) to induce cellular senescence. MAFs were cultured at 3% O_2_.

### 4.7 Tissue histology and morphometric analysis

Tissues were fixed in 10% formalin and embedded in paraffin. For lung structural analysis, sections of the left lung were cut at 5 μm and stained with H&E, as previously described (Birch et al., 2015). Airspace size was determined on 10 randomly captured images for each animal by the mean linear intercept method as described (Birch et al., 2015) using ImageJ analysis software. Epidermal thickness was determined on H&E sections (5 μm) of skin collected from the dorsal region of the animal using ImageJ analysis software, as previously described (Jurk et al., 2014).

Membrane staining for cardiac hypertrophy quantification was performed with wheat germ agglutinin (WGA) labelling (Alexa Fluor® 647 conjugate, W32466, Invitrogen) as per manufacturer's instructions. Cardiomyocyte hypertrophy was assessed by cross‐sectional area of cell membranes labelled with WGA, and troponin C was used to identify cardiomyocytes. Only cardiomyocytes in the left ventricle free wall were analysed and only in the areas in the subepicardium region. To control for tissue orientation, only cardiomyocytes that were surrounded by capillaries all displaying a cross‐sectional orientation were analysed.

### 4.8 Crosslinked chromatin immunoprecipitation (ChIP) assay

ChIP assay was carried out using 25 μg crosslinked chromatin prepared from *nfκb1* ^−/−^ livers and lung. Briefly, the chromatin was prepared by resuspending powdered, frozen lung and liver in 10 ml cold PBS with protease inhibitors and crosslinked with 1% formaldehyde for 5 min. The reaction was stopped by adding 0.125 M glycine, cells spun and pellet resuspended in lysis buffer (1% SDS, 10 mM EDTA, 50 mM Tris–HCl (pH 8.1)) for 20 min before sonication for 10 min (10 cycles of 30 s on, 30 s off) in Diagenode Bioruptor. The sonicated material was spun down, and chromatin‐containing supernatant was collected. 25 μg of chromatin was then incubated with 5 μg of anti‐p65 antibody (sc109x, Santa Cruz) or control antibody (ab46540, Abcam) overnight at 4°C. The complexes were collected using 50 μl of blocked Staph A membranes, which were then serially washed, complexes eluted off and crosslinks reversed. Genomic DNA was purified and used in quantitative PCR with mIL‐6 primers. Each PCR was performed in triplicate, and the analysis was repeated at least three times from independent ChIP experiments. A signal intensity value for each sample was calculated from the average of the experiments. Average values of eluates were normalized to average values of control antibody sample and expressed as fold enrichment above background. PCR amplification was carried out using the primers forward primer 5′‐AAGCACACTTTCCCCTTCCT‐3′ and reverse primer 5′‐TCATGGGAAAATCCCACAT‐3′.

### 4.9 Sen‐β‐Gal assay

Analysis of Sen‐β‐Gal activity in MAFs was carried out as previously described (Jurk et al., 2014).

### 4.10 Immunocytochemistry

Analysis of Ki67 positivity in MAFs was performed as previously described (Jurk et al., 2014). Briefly, MAFs were washed with PBS and fixed for 10 min with 2% paraformaldehyde. Cells were permeabilized for 45 min, and the primary antibody (anti‐Ki67 no. ab15580, rabbit polyclonal, Abcam, 1:50) was applied overnight at 4°C, followed by incubation with Alexa Fluor secondary antibody (1:2,000; Molecular Probes) for 30 min at room temperature. Cells were stained with DAPI and mounted in VECTASHIELD Mounting Media.

### 4.11 Western blotting

Western blotting was carried out on protein isolated from MAFs as previously described (Jurk et al., 2014). Primary antibodies against phospho‐S6 (Ser235/236) (Rabbit monoclonal no. 4858, Cell Signalling, 1:1,000), S6 (Rabbit monoclonal no. 2217, Cell Signalling, 1:1,000), p21 (Rabbit monoclonal no. 2947, Cell Signalling, 1:1,000) and β‐tubulin (Rabbit polyclonal no. 2146, Cell Signalling, 1:2,000) were applied overnight at 4°C. Goat anti‐rabbit IgG‐HRP conjugated secondary antibody (A0545, Sigma‐Aldrich) was applied for 1 hr at room temperature (1:5,000).

### 4.12 Immunohistochemistry (IHC)

IHC was performed and analysed as previously described (Birch et al., 2015). Antibodies against CD68 (Polyclonal Antibody (1:100, aviva system biology OABB00472)) and α‐SMA were applied overnight at 4°C. Staining was analysed with a NIKON ECLIPSE‐E800 microscope, and images were captured with a Leica DFC420 camera using the LAS software (Leica). Staining was quantified in a blinded fashion on 10 random images captured per tissue section.

### 4.13 Immuno‐FISH

For analysis of telomere‐associated foci, immunofluorescence coupled with telomere‐fluorescence in situ hybridization (immuno‐FISH) was performed. Tissues were stained and analysed as described in Hewitt et al. (2012). Briefly, anti‐γH2A.X (S139) (no. 9718, Rabbit monoclonal, Cell Signalling, 1:200) immunofluorescence was carried out followed by crosslinking tissues with 4% formaldehyde for 20 min. Tissues were denatured for 10 min at 80°C in hybridization buffer (70% formamide), 25 mM MgCl_2_, 0.1 M Tris (pH 7.2), 5% blocking reagent (Roche)) containing 2.5 μg ml Cy‐3‐labelled telomere‐specific (CCCTAA) peptide nucleic acid probe (Panagene), followed by hybridization for 2 hr at room temperature in the dark. In‐depth Z‐stacking was used (a minimum of 40 optical slices with ×100 objective) followed by Huygens (SVI) deconvolution.

### 4.14 Quantitative RT‐PCR

Q‐PCR analysis was performed as previously described in Correia‐Melo et al. (2016). mRNA expression levels were normalized to the expression of the 18S gene. Q‐PCR was run in triplicate using the following primers:

p21 Fw: 5′‐CCTGGTGATGTCCGACCTG‐3′
p21 Rv: 5′‐CCATGAGCGCATCGCAATC‐3′
p16 Fw: 5′‐TTGCCCATCATCATCACCT‐3′
p16 Rv: 5′‐GGGTTTTCTTGGTGAAGTTCG‐3′
18S Fw: 5′‐CGGCTACCACATCCAAGGAA‐3′
18S Rv: 5′‐AGCCGCGGTAATTCCAGC‐3′
IL‐6 Fw: 5′‐TGATTGTATGAACAACGATGATGC‐3′
IL‐6 Rv: 5′‐GGACTCTGGCTTTGTCTTTCTTGT‐3′

### 4.15 Cytokine array

Blood serum was collected from wild‐type and *nfκb1* ^−/−^ mice at 9.5 months of age. Senescence was induced in wild‐type and *nfκb1* ^−/−^ MAFs by X‐ray irradiation (10 Gy). At day 9 (with or without irradiation), cells were cultured in serum‐free media for 24 hr and media were collected for analysis.

Both media and blood serum were analysed for expression of several mouse cytokine and chemokines (MD31) using a Multiplexing LASER Bead Assay (Eve Technologies).

### 4.16 Sirius red staining

Sirius red staining was carried out on 10% formalin‐fixed, paraffin‐embedded liver sections (5 μm) to assess collagen deposition. Briefly, sections were dewaxed in Histoclear and rehydrated through serial ethanol dilutions. Sections were treated with 0.2% phospho molybdic acid for 5 min followed by incubation with Picro‐Sirius Red for 2 hr at room temperature. Slides were washed with 0.01% HCl and dehydrated through graded ethanol solutions. Slides were mounted with DPX and imaged using a Leica DFC420 camera using the LAS software (Leica) connected to a NIKON ECLIPSE‐E800 microscope. Staining was quantified in a blinded fashion on 10 random images captured per section.

### 4.17 Mitochondrial ROS measurements

Mitochondrial superoxide levels were measured in MAFs by flow cytometry by incubation with 5 μM MitoSOX probe (Molecular Probes, Invitrogen) as previously described (Passos et al., 2007).

Mitochondrial hydrogen peroxide release in isolated mitochondria from liver was measured fluorometrically in the presence of exogenous superoxide dismutase (15 U/ml), horseradish peroxidise (2 U/ml) and Amplex Red (50 mM) at 37°C, and the fluorescence signals were calibrated against a hydrogen peroxide standard as previously described in Miwa et al. (2014).

### 4.18 Statistical analyses

One‐way ANOVA, two‐tailed *t* test, Wilcoxon–Mann–Whitney and Kruskal–Wallis test were assessed using GraphPad Prism Version 6.0. Linear and non‐linear regression analysis test and Kaplan–Meier survival curves were conducted using Sigma Plot versus 11.0.

## 5 ETHICS STATEMENT

All work complied with the guiding principles for the care and use of laboratory animals. The project was approved by the Faculty of Medical Sciences Ethical Review Committee, Newcastle University. Project license number 60/3,864.

## CONFLICT OF INTEREST

The authors declare to have no competing financial interests.

## AUTHOR CONTRIBUTIONS

CCM and JB performed majority of experiments, designed, wrote and critically reviewed the manuscript. EF carried out behavioural and frailty assessments in animals. BMC assisted with cell culture experiments and conducted WB. SM carried out Amplex Red analysis. DR, JT, JC and AL conducted lung tissue analysis, liver analysis and skin structural measurements. GR carried out heart morphometric measurements. DJ isolated MAFs from *nfκb1* ^−/−^ mice. FO conducted histopathological analysis of the liver. JM carried out ChIP analysis. DAM provided *nfκb1* ^−/−^ mice. VIK supervised parts of the study. JFP designed experiments, wrote and critically reviewed the manuscript.

All authors read and commented on the manuscript.
