# Supplementary material for: Rapamycin improves healthspan but not inflammaging in nfκb1 −/− mice
Source: Aging Cell. 2018 Nov 23;18(1):e12882. doi: 10.1111/acel.12882 (PMC6351839; doi:10.1111/acel.12882)
Supplement: Supplementary file 1 [file ACEL-18-e12882-s001.pdf]

Supplementary data

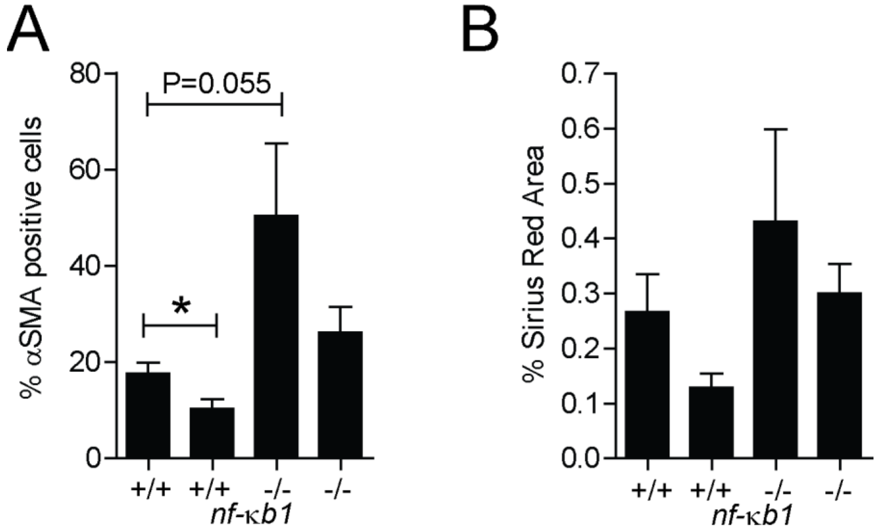

Supplementary Figure 1 - Rapamycin does not ameliorate fibrosis-associated markers in nfkb1$^{-/-}$ mice

A) Quantifications of α-SMA positive cells in the livers of wt or *nfkb1*$^{-/-}$ mice fed a control or rapamycin-supplemented diet at 9.5 months of age. B) Quantifications of sirius red staining in the livers of wt or *nfkb1*$^{-/-}$ mice fed a control or rapamycin-supplemented diet at 9.5 months of age. Data represent group mean + SEM (n=5 mice per group for all analyses). * p < 005, ** p < 0.01, ***p < 0.001. Statistics were performed using one way ANOVA followed by Tukey's multiple comparison test.

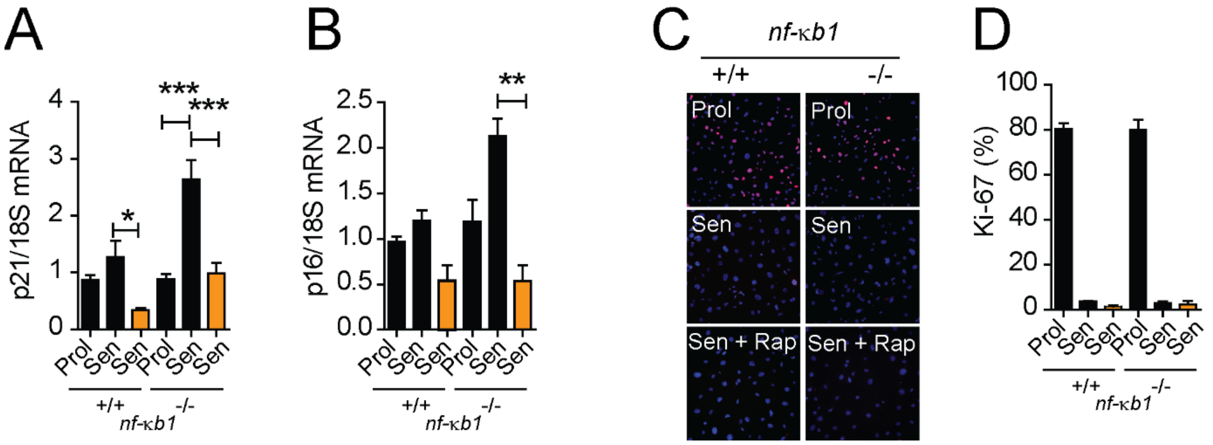

Supplementary Figure 2 Rapamycin reduces mRNA expression of cyclin-dependent kinase inhibitors p21 and p16 without resuming proliferation in senescent MAFs derived from nfkb1$^{-/-}$ mice

A) p21 and p16 (B) mRNA levels in proliferating and senescent MAFs from wild-type or *nfkb1*$^{-/-}$ mice treated with (orange) or without (black) rapamycin, normalised to 18S; C) Representative images of Ki67 positivity in proliferating and senescent MAFs from wild-type or *nfkb1*$^{-/-}$ mice treated with or without rapamycin, with quantifications on the right (D). Data represent group mean + standard error of the mean (SEM) (n=3 mice per group). * p < 005, ** p < 0.01, ***p < 0.001. Statistics were performed using one way ANOVA followed by Tukey's multiple comparison test.
