# Supplementary material for: Rapamycin improves healthspan but not inflammaging in nfκb1 −/− mice
Source: Aging Cell. 2018 Nov 23;18(1):e12882. doi: 10.1111/acel.12882 (PMC6351839; doi:10.1111/acel.12882)
Supplement: Supplementary file 2 [file ACEL-18-e12882-s002.docx]

| Table 1 \| Clinical Frailty Index (CFI) in ageing *nfkb1* ^-/-^ mice | | | | | | |
| --- | --- | --- | --- | --- | --- | --- |
|  |  | ***15 months*** | | | ***18 months*** | |
| CFI Parameter/Phenotype |  | **Control** | **Rapamycin** | | **Control** | **Rapamycin** |
|  |  | *mean score*  (n=10) | *mean score*  (n=11) | | *mean score*  (n=8) | *mean score*  (n=8) |
| *Body weight* |  | 30.58 | 31.60 | | 28.05 | 28.11 |
| *Body surface temp* |  | 28.41 | 28.35 | | 26.91 | 27.98 |
|  |  |  |  | |  |  |
| *Integument* |  |  |  | |  |  |
|  | Alopecia | 0.05 | 0.00 | | 0.44 | 0.13 |
|  | Loss of fur colour | 0.40 | 0.5 | | 0.63 | 0.38 |
|  | Dermatitis | 0.00 | 0.00 | | 0.00 | 0.00 |
|  | Loss of whiskers | 0.35 | 0.23 | | 0.19 | 0.00 |
|  | Coat condition | 0.15 | 0.36 | | 0.38 | 0.38 |
| *Physical/*  *Musculoskeletal* |  |  |  | |  |  |
|  | Tumours | 0.00 | 0.00 | 0.00 | | 0.00 |
|  | Distended abdomen | 0.00 | 0.00 | 0.00 | | 0.00 |
|  | Kyphosis | 0.00 | 0.05 | 0.44 | | 0.25 |
|  | Tail stiffening | 0.00 | 0.00 | 0.44 | | 0.38 |
|  | Gait disorder | 0.20 | 0.00 | 0.13 | | 0.00 |
|  | Tremor | 0.00 | 0.05 | 0.06 | | 0.00 |
|  | Forelimb-grip strength | 0.25 | 0.18 | 0.13 | | 0.00 |
|  | Body condition score | 0.10 | 0.13 | 0.06 | | 0.06 |
| *Ocular/Nasal* |  |  |  | |  |  |
|  | Cataracts | 0.00 | 0.00 | | 0.00 | 0.00 |
|  | Corneal Opacity | 0.00 | 0.00 | | 0.00 | 0.00 |
|  | Eye discharge/swelling | 0.00 | 0.05 | | 0.06 | 0.00 |
| *Digestive/*  *Urogential* |  |  |  | |  |  |
|  | Malocclusions | 0.00 | 0.00 | | 0.00 | 0.00 |
|  | Rectal prolapse | 0.00 | 0.00 | | 0.00 | 0.00 |
|  | Vaginal/uterine/penile prolapse | 0.00 | 0.00 | | 0.00 | 0.00 |
|  | Diarrhoea | 0.00 | 0.00 | | 0.00 | 0.00 |
| *Discomfort* |  |  |  | |  |  |
|  | Mouse grimace scale | 0.10 | 0.00 | | 0.25 | 0.13 |
|  | Piloerection | 0.25 | 0.32 | | 0.31 | 0.56 |
|  |  |  |  | |  |  |
| *Mean score Sum ** | | 0.10 | 0.09 | | 0.19 | 0.10 |
| The Clinical Frailty Index (CFI) was evaluated in 15 and 18 months old *nf-kb1* -/- mice fed with rapamycin or control diet from the age 4 months. Data was obtained by rating each CFI parameter/phenotype as 0 = absent 0.5 = mild 1 = severe and are presented as CFI mean score per animal group for the indicated parameter/phenotype. Lower CFI mean scores reflect milder ageing-associated phenotypes.  * Mean score Sum excludes body weight and body surface temperature mean scores. | | | | | | |

**Table 1 Clinical frailty Index in ageing *nfkb1^-/-^* mice fed control or rapamycin-supplemented diet.**

| Table 2 \| Liver histopathology of *nf-kb1^+/+^* vs  *nf-kb1 ^-/-^* mice | | | | | | | | |
| --- | --- | --- | --- | --- | --- | --- | --- | --- |
|  |  | | **9.5 months** | | | | **Moribund** | |
|  |  | | *nf-kb1 ^+/+^* | | *nf-kb1 ^-/-^* | | *nf-kb1 ^-/-^* | |
| *Histopathology* | |  | **Control** | **Rapamycin** | **Control** | **Rapamycin** | **Control** | **Rapamycin** |
|  | |  |  |  |  |  |  |  |
| *Inflammation* | |  | 2/5 | 0/5 | 4/4 | 4/5 | 7/8 | 7/7 |
| *Fibrosis* | |  | 0/5 | 0/5 | 0/4 | 0/5 | 3/8 | 4/7 |
| *Dysplasia* | |  | 0/5 | 0/5 | 0/4 | 0/5 | 1/8 | 3/7 |
|  |  | |  |  |  |  |  |  |

**Table 2 Liver histopathology in wt and *nfkb1^-/-^* mice fed control or rapamycin-supplemented diet.** Data are number of animals showing the phenotype/number of animals observed.
